# Mechanotransduction: A Master Regulator of Alveolar Cell Fate Determination

**DOI:** 10.3390/bioengineering12070760

**Published:** 2025-07-14

**Authors:** Kusum Devi, Kalpaj R. Parekh

**Affiliations:** Department of Cardiothoracic Surgery, Carver College of Medicine, University of Iowa, Iowa City, IA 52242, USA; kusum-devi@uiowa.edu

**Keywords:** mechanotransduction, cell fate maintenance, AT-AT2 differentiation, mechanotransduction-mediated signaling

## Abstract

Mechanotransduction plays an essential role in the fate determination of alveolar cells within the pulmonary system by translating mechanical forces into intricate biochemical signals. This process exclusively governs differentiation, phenotypic stability, and maintenance of alveolar epithelial cell subtypes, primarily the alveolar AT1/AT2 cells. Perturbed mechanical tension proportionally impacts alveolar cell phenotypic identity and their functional characteristics. The fundamental influence of respiratory mechanics on alveolar cell lineage commitment and sustenance is undeniable. AT1 cells are recognized as principal mechanosensors within the alveolus, directly perceiving and responding to mechanical forces imposed by respiration through cell–matrix interactions. These mechanical forces instigate a profound reorganization of the actin cytoskeleton within cells, indispensable for signal transduction and perpetuation of their differentiated phenotype, orchestrated by integrins and cell adhesion molecule-mediated signaling. The dysregulated mechanotransduction in the pulmonary system intrinsically contributes to the etiology and progression of various diseases, exemplified by pulmonary fibrosis. This review systematically elucidates the profound impact of mechanotransduction on alveolar cell differentiation and fate sustenance and underscores how its dysregulation contributes to the initiation and perpetuation of lung diseases.

## 1. Introduction

Mechanotransduction is an essential mechanism that supports biological function in organisms. Primarily, it was explored relying on Wolff’s law of mechanical loads in tissue homeostasis and extensively studied in growth and development [1]. In the last decade, the crucial mechanical impact of cellular mechanotransduction has been investigated in various physiological events such as embryonic development [2], tissue repair [3], tumorigenesis [4], fibrosis [5], neural regeneration [6], and immunotherapy resistance [7]. Mammalian lungs develop to be elastic and flexible to facilitate the effective exchange of gases by allowing alveoli to sustain periodic inflation and deflation. The alveolar niche is made up of several cell lineages, such as different mesenchymal, endothelial, alveolar type 1 (AT1), and (AT2) epithelial cells. The majority of the research on lung mechanotransduction is centered on the responsiveness of mesenchymal lineages and their capacity to develop into myofibroblasts in conditions like IPF [8]. Recent findings have demonstrated the impact of biophysical force mediated by respiration in the fate decision of AT1 and AT2 cells in the lungs. Respiratory forces dynamically orchestrate alterations in nuclear lamina–chromatin interactions to preserve AT1 cell identity and limit their reprogramming into AT2 cells [9]. Indeed, recent breakthroughs in the realm of molecular biology have rendered it possible to investigate the molecular aspects in ever-greater detail. The enormous amount of data generated by molecular approaches suggests that determining the fate of individual cells is a challenging and intricate process. To resolve the complex interplay between the biochemical and molecular regulation of cell fate determination, researchers have also examined the impact of physical forces that result in the morphogenesis of the cells, tissue, organs, and organisms. This review discusses the mechanism and provides a better understanding of mechanical forces within biological systems to determine lung alveolar cell fate.

## 2. Roadmap of Mechanotransduction

Mechanotransduction is a biological phenomenon in which cells perceive one or more external forces and respond biochemically by changing their phosphorylation state, translocating proteins, or modifying conformational changes [1,10]. In certain situations, this can also result in gene regulation, transforming biological outcomes like cellular phenotype, migration, and/or behavior. Subsequently, it has been anticipated that mechanotransduction will commence an assortment of biological processes, notably tissue regeneration, repair, and development in the embryo. On the other hand, pathogenic processes like multi-organ fibrosis, carcinogenesis, and cancer immunotherapy resistance can be driven by sustained, excessive mechanical stimulation [10]. In the past few decades, mechanotransduction has been explored experimentally as well as theoretically, which has allowed researchers to define and identify some mechanotransduction components known as mechanosensors more precisely. Researchers have also determined the nature and provenance of physical cues emanating from biological systems. The research investigating embryogenesis has revealed that several developmental processes, including tissue growth, migration, and rearrangement, are influenced by external or intrinsic mechanical stimuli, including tensile forces, fluid shear stress, and hydrostatic pressure. These mechanical inputs govern fluid-to-jamming transition, as indicated for body axis elongation, or by enhancing the head mesoderm’s cell density. Additionally, tissue compaction in mice is one example of how these mechanical inputs regulate cellular development [11,12].

**Figure 1:**
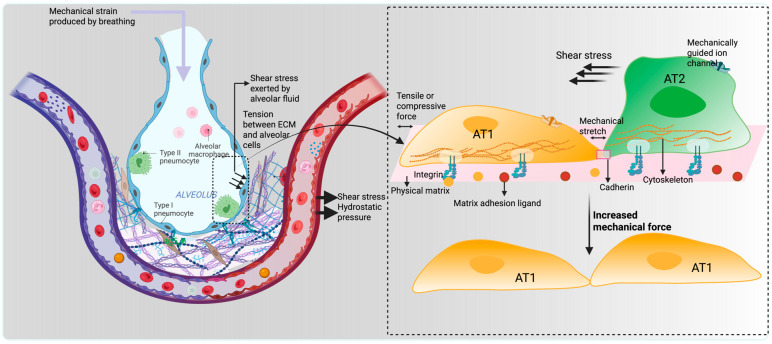
Mechanical strain is applied to the alveolus during the breathing process, and hydrostatic, shear, and tensile forces are experienced by the alveolar cells depending on their locations. Alveolar cells directly exposed to the mechanical strain differentiated into AT1 cells via stretch-mediated signaling, while other cells underwent transdifferentiation into AT2 cells.

In alveolar mechanotransduction, stress refers to the force per unit area applied to alveolar tissue or cells. This can arise from air or fluid pressure within the alveoli or from external forces such as mechanical ventilation [13]. Physical strain is defined by the proportional alteration in the length or shape of an object in response to a force applied to it. Moreover, stretch specifically describes the mechanical distension or elongation that alveolar cells undergo, particularly during lung inflation and deflation. In the context of alveolar cells, stretch is often used interchangeably with strain but typically refers to the actual physical expansion experienced by the cells, especially at the basolateral surface, due to basement membrane expansion during breathing [14]. Breathing continuously exerts mechanical strain on alveolar epithelial cells; however, sheer stress, strain, and hydrostatic pressure primarily affect the vascular endothelial cells. Depending on their location, various lung cell types respond to different mechanical forces. For instance, the fluid layer in the alveoli and airways generates shear stress on the apical surface of the epithelium. At the same time, the expansion of the basement membrane causes strain on the epithelium basolateral surface. Thus, two distinct “mechanical forces” are applied to the same cell type to govern different biological processes by enhancing cytoskeleton reorganization and nuclear signaling. For example, mechanical strain primarily mediates epithelial cell proliferation, migration, and differentiation [15], and sheer stress impacts barrier integrity and permeability [16,17]. A centripetal force is applied to the surrounding matrix by the interconnected biopolymer network known as the cytoskeleton, which is located within the plasma membrane. Strain generated through actin cytoskeleton contraction extends to the entire cell, including the nucleus. Adhesion receptors like integrins, which serve as a bridge between the cytoskeleton and the extracellular matrix, permit pulmonary cells to communicate with their surroundings. Mechanical forces beyond physiological limits can cause cellular barrier breakdown, metabolic dysfunction, and ignite inflammation [18,19]. Excessive or aberrant mechanical tension alters AT2 cell behavior, leading to impaired proliferation and defective differentiation into AT1 cells, which are crucial for restoring the alveolar surface after injury. For instance, increased mechanical tension after alveolar loss promotes actin polymerization and YAP activation in AT2 cells, but when dysregulated, this can result in spatially restricted fibrosis rather than effective regeneration [20] (Figure 1).

## 3. Mechanotransduction Forces Govern Alveolar Cell Development and Differentiation

The core physiologic function of the lung is vital gaseous exchange, ensuring a constant supply of life-sustaining oxygen to the body and the efficient removal of metabolic waste in the form of carbon dioxide [21]. This process is driven by the diffusion of gases across a shared basement membrane, a delicate interface between the expansive AT1 cells and a unique group of endothelial cells distinguished by their expression of carbonic anhydrase 4 (CAR4) [22]. AT1 cells are remarkably elongated and thin cells that are visualized as bedsheets draping over the other cells [23,24,25]. AT1 cells are considered paramount mechanosensors in the alveolus, and they directly perceive and respond to mechanical forces from respiration through their cell matrix [9]. Unlike their counterparts, AT2 cells are compact and cuboidal yet play a vital role by secreting surfactants essential to maintaining surface tension and preventing regional alveolar atelectasis [26,27]. These activities of AT2 cells are governed by regulated mechanical forces. AT2 cells are directly exposed to the mechanical forces generated by cyclic stretch from breathing. These forces are sensed and respond through various mechanotransduction pathways and stimulate key biological functions of AT2 cells, including proliferation, surfactant secretion, and differentiation into AT1 cells [17]. Additionally, distinct fibroblast populations within the alveolar interstitium hint at a complex interplay of cellular signals that orchestrate lung function. Myofibroblasts generate the mechanical forces required for septal ridge formation and alveolar remodeling through actomyosin-mediated contraction. Disruption of their contractile function leads to alveolar simplification and impaired lung function [28]. Additionally, myofibroblasts respond to Shh ligands from AT1 epithelial cells, and inhibition of Shh signaling during critical developmental windows disrupts myofibroblast expansion and impairs alveolarization [29]. Myofibroblasts are indispensable for alveolar cell development by generating contractile forces, producing ECM, supporting epithelial cell proliferation, and integrating key developmental signals. Their coordinated activity ensures the formation of a structurally complex and functional alveolar network [30]. Alveolar macrophages, strategically positioned within the alveoli and around blood vessels, likely exhibit a multifaceted role in regulating immune defense and interstitial processes [31]. Like other lung cells, alveolar macrophages are sensitive to mechanical cues in their environment, such as changes in substrate stiffness, cyclic stretch, and tissue remodeling during development and disease [32]. During lung development, the dynamic mechanical environment, shaped by breathing movements, tissue expansion, and matrix remodeling directly influences macrophage activation and function. Mechanically activated macrophages can modulate the local microenvironment by secreting growth factors and cytokines that influence alveolar epithelial cells proliferation, differentiation, and repair. When exposed to stiffer substrates or increased mechanical stress, alveolar macrophages display altered morphology, increased pro-inflammatory cytokine production (e.g., TNF-α, IL-6), and enhanced cell–matrix adhesion and motility [33]. They sense these mechanical signals through specialized pathways involving integrins, mechanosensitive ion channels (e.g., TRPV4, PIEZO1), and signaling mediators such as YAP/TAZ [32].

This diverse landscape of cells exhibits a spectrum of varied abilities to withstand mechanical forces during respiration [34]. Lungs are constantly stretched and compressed during breathing, which creates mechanical forces on the cells in the alveoli, and how lungs maintain cellular homeostasis under continuous bio-physical stress is still elusive. Mesenchymal cell sensitivity is erudite to mechanical forces, while epithelial cells are less well understood in this regard. This is an area of ongoing research, and scientists are working to obtain improved, comprehensive knowledge of how epithelial cells perceive and respond to mechanical forces.

Classically, alveolar cells develop into two different phenotypes, AT1 and AT2. AT2 cells can develop into AT1 cells, which are primarily involved in gas exchange and do not exhibit any proliferative capacity. More than 95% of the alveolar surface area is comprised of AT1 cells, which modify paracellular permeability in response to periodic stretches by altering their genetic makeup. Mechanical events promote the proliferation, secretion, metabolism, cellular apoptosis [35], and migration of AT2 cells [15,36]. The maintenance of the epithelium integrity is contingent upon the proliferation of alveolar epithelial cells, particularly during the reparative phase following lung injury. Alveolar epithelial cells undergo mitosis and secrete growth factors in response to mechanical stimuli. When exposed to conditioned media from lung fibroblasts, AT2 cells that are periodically treated to mechanical stress in FlexCell units exhibit enhanced chromatin synthesis in comparison to those that develop under static circumstances. During lung development, mechanical stress also stimulates the PDGFRB expression [37]. Cyclic stretch exerted during fetal breathing helps fetal lung cells to increase PDGF-B gene expression and PDGF-BB protein levels, and this effect is critical for promoting lung cell proliferation and proper lung growth. Additionally, mechanical stress augments PDGFRB expression and its downstream signaling in pulmonary artery tissue and smooth muscle cells, further supporting its role in lung development under mechanical cues. Cyclic stretch gradually escalates intracellular calcium ion concentrations, which causes the AT2 cells to release surfactant phospholipids [38]. The inhalation of amniotic fluid by fetal breathing movements generates a strong mechanical force, which is crucial for cell differentiation in lung development [39]. Recent findings have detected the stretch-activated currents in E17.5 mouse AT2 cells and showed the role of mechanosensitive TMEM63A and TMEM63B channels in stimulating stretch-mediated surfactant release. It was additionally claimed that the deficiency of TMEM63A/B channels might trigger decreased alveolar surfactant levels and lead to breathing failure after birth [38].

## 4. Mechanical Force Intensity Regulates the Differentiation of Alveolar Epithelial Cells

**Figure 2:**
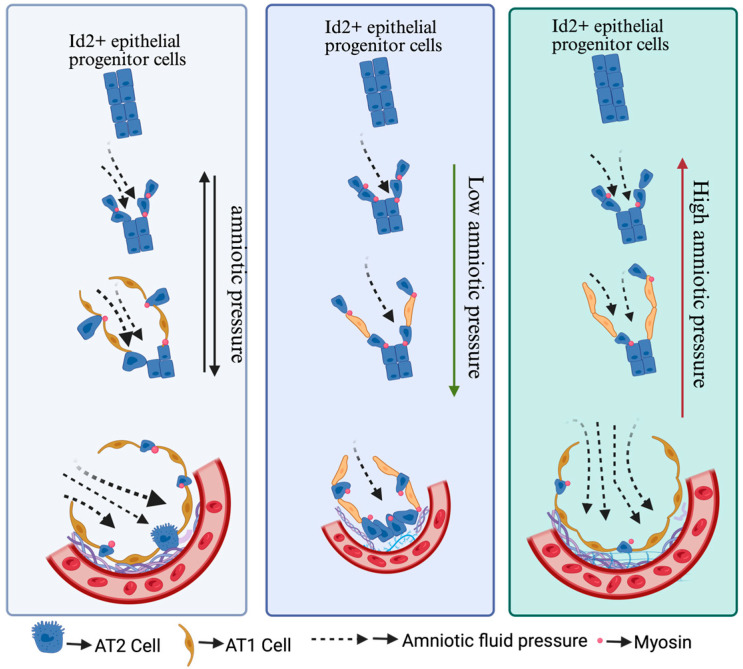
AT1/AT2 differentiation is correlated with the fluid pressure in growing airspaces, particularly throughout late prenatal development. Lower fluid pressure led to more AT2 cell differentiation, while higher fluid pressure encouraged the differentiation of AT1 cells. There is a notable possibility that elevated pressures on the growing epithelium might encourage AT1 cell fate-specific proteins, such as YAP, to translocate into the nucleus due to stretch, supporting AT1 cell development and overriding earlier developmental events related to cellular specification.

AT1/AT2 cell differentiation is a crucial process for lung development and is essential for maintaining optimal gaseous exchange. Abnormality in AT1/AT2 cell differentiation can trigger severe consequences, leading to a range of respiratory problems, including pulmonary fibrosis, emphysema, and lung cancer [40,41,42]. Researchers have demonstrated that fetal breathing movements drive amniotic fluid inhalation, which generates mechanical forces crucial for the differentiation of AT1 cells. These mechanical forces act as a key regulator that influences the differentiation trajectory of alveolar progenitor cells. A significant portion of these progenitor cells extend protrusions from the airway epithelium into the mesenchyme. This protrusion process is dependent on signaling via fibroblast growth factor 10 and FGF receptor 2 (FGF10/FGF2). The formation of these protrusions leads to myosin aggregation at the apical surface of protruding cells [43]. This apically concentrated myosin provides structural reinforcement, enabling the cells to withstand the mechanical forces imposed by the inhaled amniotic fluid (Figure 2). This resistance, in turn, facilitates the maintenance of the cuboidal morphology characteristic of these progenitor cells. AT2 cells have a limited alveolar epithelium surface area, and this, coupled with their apparent resistance to mechanical stress, suggests a specialized adaptation for survival in the dynamic lung environment. Hence, this myosin-mediated contractility ensures the protruding AT2 cells fate by preventing them from becoming flattened under the influence of mechanical forces. It also manifests that adult AT2 cells are superior at withstanding the mechanical force compared to AT1 cells [39]. Moreover, non-protruded alveolar progenitors differentiate into AT1 cells in the influence of fatal breathing mechanical forces at E17. Further results have demonstrated that FGF10 secreted by mesenchymal cells directs the Id2+ progenitor cell migration, proving that FGF10 is epitomized as a local chemotactic factor to trigger actin-based cell protrusion process. Moreover, they are synergistically controlling cell differentiation. In essence, mechanical cues shape cell morphology and fate, while growth factors provide directional guidance and proliferative signals, working in concert to orchestrate the complex process of alveolar development [39].

## 5. Chemical and Mechanical Cues Interplay in Alveolar Cell Fate Determination

Mechanotransduction is a biological phenomenon in which cells perceive one or more external physical forces and respond biochemically by changing their phosphorylation state, translocating proteins, or modifying conformational changes. In particular instances, it also governs gene regulation, resulting in altered biological outcomes like cellular phenotype, migration, and/or behavior. Undoubtedly, mechanotransduction transforms the physical forces into biochemical responses at a specific point to execute the biological process in the system. Researchers have identified various intrinsic and extrinsic mechanical and biophysical cues responsible for alveolar cell fate decisions during development [44]. AT1 cells are responsible for gaseous exchange in the lungs and are considered terminally differentiated, readily becoming reprogrammed into AT2 cells when their cytoskeleton is disturbed. Shiraishi et al. described the role of Cdc42 and Ptk2 in AT2-AT1 cell differentiation. Small RhoGTPase Cdc42 is a critical regulator of actin cytoskeleton reorganization that is preferentially expressed in AT1 rather than AT2 cells in the adult mouse lung and is required to maintain AT1 cells’ fate during normal homeostasis in the absence of injury [45]. Loss of Cdc42 in AT1 cells leads to enhanced expression of AT2 cell marker genes, including Sftpc, suggesting rapid re-programming into AT2 cells. Single-cell sequencing data shows reprogrammed AT2 cells cluster with WT AT2 cells and express AT2 cell-specific genes at a similar level to WT AT2 cells. The Cdc42-dependent rearrangement of the actin cytoskeleton contributes to the active maintenance of AT1 cell identity, prohibiting the reprogramming of these cells into AT2 cells [9]. Additionally, Ptk2 deletion in AT2 reduces the ability of AT2 to differentiate in AT1 cells in Ptk2^AT2-KO^ mice compared to control mice. Importantly, loss of Ptk2 in AT1 cells simultaneously reprogrammed into AT2 cells, suggesting that Ptk2 is essential for AT1 cell fate maintenance. Collectively, these data demonstrate that transduction of extracellular mechanical forces into the cell through integrin signaling is required for AT2–AT1 cell differentiation and AT1 cell fate maintenance [9].

Additionally, it has been reported that transmural fluid-mediated pressure determines cell differentiation in early and late mouse lung organogenesis. Early embryonic lungs that were successfully isolated and cultured showed branching and growth like those of embryos developing under high transmural pressure. On the other hand, low transmural pressure is associated with aberrant pulmonary development and branching [46]. Fluids are also translocated toward the branching tips of the lung during fetal breathing [39]. AT2 cells develop when the alveolar progenitor, particularly residing in the branching terminals, limits their apical surfaces to protect them from pressure; in contrast, neighboring cells experiencing hydrostatic pressure develop thin, elongated AT1 cells [39]. The fate outcome of alveolar cells synergistically depends on the sensing pressure and growth factors (FGF10/FGFR2). Importantly, FGF10 triggers ERK signaling, which results in the formation of protrusions that shield alveolar progenitors from pressure. The inhibition of FGF10/FGFR2-mediated actin-based cell protrusion showed nearly all the basal buds are flattened and showed AGER+ cells instead of SftpC. Thus, it leads to considerable impairment in AT2 cell differentiation, indicating the significance of the FGF10 pathway in AT2 fate determination [39]. A recent finding elaborated the crucial relationship between ROCK-YAP/TAZ signaling in the AT1 cell differentiation. The significance of this pathway is evident, as stretch is correlated with the nucleocytoplasmic shuttling of Yap/Taz, predominantly in AT1 cells. It additionally increases the number of these AT1 cells. These effects were further reversed after the application of ROCK. From the ex vivo model, it was evident that the ROCK inhibitor Y27632 affected Yap/Taz activation because its addition is associated with a decreased nuclear Yap/Taz-positive cells in both the stretched and control groups. Moreover, a declining trend in the number of AT1 cells was seen upon Y27632 addition [47]. Collectively, YAP/TAZ are central regulators of alveolar epithelial differentiation, acting as mechanosensitive transcriptional co-activators that integrate mechanical and biochemical signals to drive AT2-to-AT1 cell differentiation, support alveolar repair, and maintain lung epithelial homeostasis.

## 6. Types of Mechanical Cues Responsible for Alveolar Cell Development in the Lung

Mechanical cues are categorized into intrinsic and extrinsic stimuli, and both are recognized as mediating cellular differentiation. ECM stiffness, cell shape, and topography are examples of intrinsic cues, whereas extrinsic cues are defined as externally applied forces such as fluid flow, hydrostatic pressure, and tension [48]. The mechanical landscape within the organism is intricate; extrinsic and intrinsic forces are interdependent and cannot be decoupled. Mechanical cues are perceived by mechanosensitive elements, e.g., membrane channels (piezo1/2) [1], adhesion molecules [48], cytoplasmic proteins, intracellular components [49], and, more importantly, the nucleus acts as a crucial mechanosensor in the cells to transmit the signal [50]. These signals prompt the cytoskeleton to respond to activated mechanical cues by altering contractility to counterbalance the forces [51]. Variations in cytoskeletal tension can trigger signaling cascades that result in transcriptional modifications that govern cellular behavior, including decisions on the destiny of individual cells. In addition, direct interactions between the nucleus and cytoskeleton are crucial for mechanotransduction [52]. Nevertheless, when the cytoskeleton is compromised, this barrier is hindered. Therefore, mechanotransduction is not a “one-way street” signal from the nucleus, it could be reverse transmitted to the cytoskeleton, resulting in a transcriptional feedback loop [53]. Previous findings have demonstrated that shear stress, a significant force imposed by flowing amniotic fluid within the epithelial air tree to the epithelium surface, controls cell fate decisions. The activation of FGF10 signaling via the surrounding stromal cells conduces to myosin accumulation at the apical surface of the protruding epithelium. The epithelium endures shear stress upon inhalation of amniotic fluid. The protruded cells inside the epithelium are shielded from mechanical stresses by the accumulated myosin, leading to the formation of AT2 cells that maintain their cuboidal shape. On the contrary, shear stress-exposed cells develop into flattened AT1 cells [39]. The mechanism of alveolar specification could be better understood by examining the reverse interplay between mechanical forces and biochemical signals.

## 7. Extracellular Matrix (ECM) Is Critically Important in Alveolar Differentiation

The ECM is well-known for participating in mechanosensitive signal transduction and influences an array of cell functions, including fate determination, cell shape, proliferation, and migration, by interacting with physical and chemical signals. From the perspective of biophysics, ECM plays an essential part in tissue’s elasticity attributes, which provides the potential to regulate different biological behaviors. The elasticity of ECM is determined by its stiffness, which is a parameter of the tissue’s resistance to external physical forces that trigger material deformation. Growing research has elucidated that embryonic ECM is dynamic, governs morphogenesis, and produces internal cues to direct tissue shape autonomously. Previous findings have demonstrated that spatial and temporal alterations in two major ECM proteins, elastin and laminin, perturbed alveolar development [54]. For instance, laminin-rich basement membranes favor maintenance of AT2 cell differentiation and promote their maturation, while fibronectin-rich matrices are often upregulated during lung injury and accelerate loss of AT2 differentiated features and promote transition toward AT1 cells [55]. Interaction of β1 integrin-mediated cell–ECM interactions establish the proper AT2/AT1 differentiation. Disruption of this interaction impairs AT2-to-AT1 differentiation, leads to excessive proliferation of intermediate cells, and results in defective alveolar repair with persistent inflammation and emphysema-like changes [56]. The precise mechanism by which elastin contributes to alveogenesis is elusive; however, developing a deeper understanding of the localization and structure of elastin fibers could assist in acquiring evidence concerning its function in alveogenesis. The 2-D images demonstrate how elastin either forms an isolated dot or patch at the tips of the “finger-like” protrusions identified as developing septa, or it partially rims the alveoli lined with AT1 [54]. Elastin localization at the tips of secondary septa suggests its involvement in driving the growth and formation of these septa during alveolarization. The formation of these septa increases the surface area for gas exchange. In essence, the location of elastin is driven by mechanical forces, indicating its crucial role in the development and maintenance of the alveolar structure and function.

## 8. Involvement of Focal Cell Adhesion Molecules and Cytoskeleton Proteins in Alveolar Differentiation and Fate Maintenance

The focal adhesion kinase (FAK) pathway is a central integrin-mediated pathway and plays an indispensable role in cellular development via actin cytoskeleton organization. It exerts mechanical cues from the external environment to the cell via integrin-mediated mechanisms [57]. AT1/AT2 show cell type-specific integrin subunits such as AT1 (integrin 5) and AT2 (integrin-6). Micropattern experiment analysis has indicated the significance of integrin-FAK signaling during cell differentiation. Moreover, lineage-labeled AT2-specific cells expressed elevated AT1-specific integrins (Itgb5), not the AT2-specific integrins (Itgb6), after acute lung injury. Escalated FAK phosphorylation was observed in the Krt8+ transition state of AT2 cells, and suppression of FAK in lineage-labeled AT2 cells exhibited impairment in AT1 differentiation. Disrupting this FAK-integrin signaling axis appears to fundamentally alter the cellular program, leading to a switch in cell phenotype from AT1 to AT2; collectively, this information demonstrates its essential participation in the alveolar cell differentiation [9].

## 9. Impact of Mechanotransduction on Nuclear Lamina–Chromatin Interactions to Direct Alveolar Cell Fates

**Figure 3:**
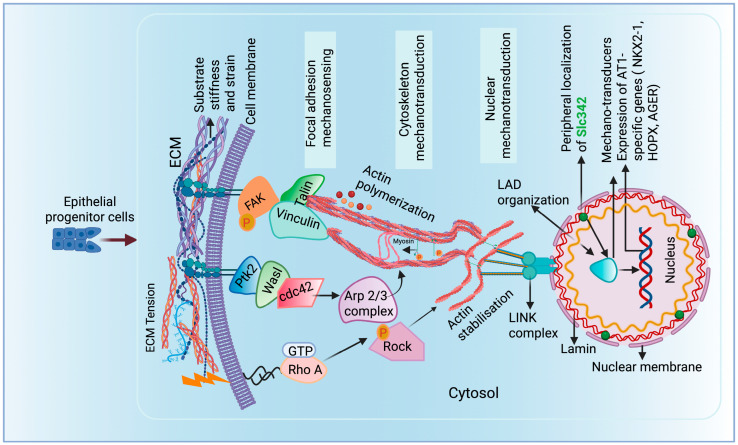
ECM is a network of tissue-specific proteins such as collagen, laminin, and fibronectin that surround cells and transmit intracellular forces. Cells attach to ECM through adhesion complexes, particularly focal adhesion proteins (FAK), comprised of integrins, talin, vinculin, and other proteins. The binding affinity of proteins can be modulated through intra- and extracellular signaling (RhoA/ROCK-mediated). Forces are transmitted along the cytoskeleton, a network of actin, microtubules, and intermediate filaments. This network connects to the nucleus and lamins, which form the nuclear structure and bind to DNA. This chain of connections allows mechanical signals from the ECM to reach the nucleus and influence gene expression, such as AT1-specific gene transcription.

The intricate and complex interplay between mechanical forces and gene expression underscores the cells’ remarkable ability to respond to the surroundings. External forces stretch the nuclear membrane, which initiates a cascade of events that reshapes the genome organization [58,59]. Large-scale physical chromatin modifications, such as the reorganization and retention of certain domains near the nuclear edge, are the most accepted concepts [60]. Lamina-associated domains (LADs) are large, transcriptionally repressed chromatin found on the nuclear edge and linked to the nuclear lamina with a multiprotein linker of the nucleoskeleton and cytoskeleton (LINC) complex [61]. Specific loci are repositioned toward and far from the lamina during in-cell state alteration, which influences the expression patterns of genes specific to a given cell lineage. AT2 LAD genes are enriched with focal adhesion proteins such as Wasl, Rock1, and Ptk2. Wasl mediates the Arp2/3 complex activation via binding with Ptk2 and governs actin polymerization [62]. The spatial location of Slc34a2 canonical AT2 marker, at the nuclear periphery of AT1 cells, activates the nuclear material rearrangement, suggesting that nuclear lamina–chromatin interactions act as a “gatekeeper” ensuring that only AT1-specific genes are active while AT2 programs are silenced through spatial genome organization. Through the repositioning of LADs in the influence of mechanotransduction, exclusively AT1-specific genes are transcribed and direct AT1 cell formation. Hence, mechanotransduction plays an essential role in AT1/AT2 cell fate maintenance via spatial genome repositioning (Figure 3).

## 10. Involvement of Ion Channels in Mechanotransduction in Alveolar Cell Differentiation

Throughout embryonic development, stretching drives lung development and maturation. Mechanical stretching activates sodium channels (ENaC) present on alveolar cells and helps to facilitate the evacuation of lung fluid during the perinatal period when the lung transitions from a fluid-filled to an air-filled organ. Proper fluid clearance is essential for alveolar expansion and maturation. Cyclic stretch during fetal breathing movements upregulate ENaC expression in alveolar epithelial cells via the p38-MAPK and JNK pathways. This increased expression enhances the sodium uptake, driving water reabsorption and facilitating lung aeration at birth [63]. Furthermore, mechanical stretching during breathing or lung inflation opens various intracellular ion channels on the alveolar cells due to increased tension on the plasma membrane [64]. TRP and Piezo are the two primary ion channels that govern the Ca^2+^ ion release in the alveolar epithelial cells. TRP channel (e.g., TRPV4) is activated by both mechanical and chemical stimuli. TRPV4, in particular, responds to physiological levels of stretch and subsequently allows the Ca^2+^ ion influx into the cell from the extracellular environment. Stretch-mediated calcium influx promotes the differentiation of AT2 into AT1 cells, facilitating the transition necessary for alveolar maturation and repair [65]. Ca^2+^ influx through Piezo and TRP channels activates pathways (such as YAP/TAZ, MAPK, and others) that regulate gene expression linked to cell differentiation [66]. Piezo1/2 are exclusively activated by mechanical stimuli such as membrane stretch or compression. Upon activation, extracellular calcium (Ca^2+^) flows into the cell, generating a rapid and reversible calcium signal [67], which leads to AT2 to AT1 differentiation. TRPV4 is primarily responsive to physiological stretch, supporting normal differentiation, while Piezo2 responds to higher, potentially injurious stretch, which can lead to cell death or pathological remodeling if unregulated [67]. The intensity and the context of mechanical stimulation determine which channels are primarily activated and how they influence alveolar cell fate decisions. Other TRP channels, such as TRPC6, are widely involved in endothelium and myofibroblast differentiation and indirectly influence the alveolar environment [68]. In ARDS patients, Piezo1 induces Bcl-2-dependent alveolar apoptosis by Ca^2+^ influx; however, these consequences have been lessened following the blocking of Piezo1 [69]. Mechanistically, mechanical straining stimulates tyrosine kinase phosphorylation to activate phospholipase Cγ via tyrosine phosphorylation [70]. Although there is currently insufficient data to conclude that these processes are directly assisting in the preservation of AT1 and AT2 cell fate, it is reasonable to assume that they have an indirect involvement in determining the fate of AT1/AT2 cells in regard to mechanical stretch during homeostasis or after injury.

## 11. Mechanosensitive Signaling Pathways Regulating AT1-AT2 Cell Differentiation

### 11.1. RhoA/ROCK Pathway

The Rho-GTPase signaling consists of a small messenger family that is tightly coupled with the cytoskeleton of the cells and is a promising option to facilitate stretch-induced cell signaling. It is universally known that the cytoskeleton is a universal mechanical force responder [71]; hence, Rho-GTP activation takes place early and upstream within the cell upon stretch. Rho GTPases actively participate in cell migration [15], lung branching morphogenesis [72,73], and, most recently, AT2 cell maturation [74]. The recent research has shown the significance of static and cyclic stretch, promoting AT1 and AT2 cell phenotypes in animal models [75]. The Rho/ROCK kinase serves remarkably in actin cytoskeleton regulation and mechanotransduction-responsive differentiation, resulting in an excellent strategy to translate cell stretch into gene expression [76]. Previous findings have provided compelling evidence that the activation of the Rho pathway remarkably regulates the static stretch-mediated differentiation of the fetal lung epithelium either via enhancing the stress fibers or proportionally by sensing GTP-bound Rho. The Rho/ROCK pathway transduces mechanical stretch into gene expression changes that promote the transition from AT2 to AT1 cells. Mechanical stretch typically suppresses AT2 markers (such as SP-B and PGC) and induces AT1 markers (HOPX, Caveolin-1, and PAI-1). Interestingly, the application of a Rho-specific inhibitor curbed the stretch-governed shifts in epithelial cell markers and maintained the AT2 markers, subsequently, the induction of AT1 markers is reduced [77]. The results from the ex vivo model demonstrated that the inhibition of ROCK using Y27632 (ROCK inhibitor) mediates a significant decline in AT1 cell population via influencing the nuclear shuttling of YAP/Taz [47]. Moreover, ROCK inhibition ameliorated actin stress fiber formation and focal adhesion plaque assembly in alveolar epithelial cells, leading to decreased cell stiffness and altered cell–matrix interactions supporting AT2 cell maintenance [78]. In essence, RhoA/ROCK inhibition regulation maintains AT2 cell marker expression and inhibits the stretch- or injury-induced upregulation of AT1 markers, thereby modulating alveolar epithelial differentiation.

### 11.2. YAP/TAZ Signaling

**Figure 4:**
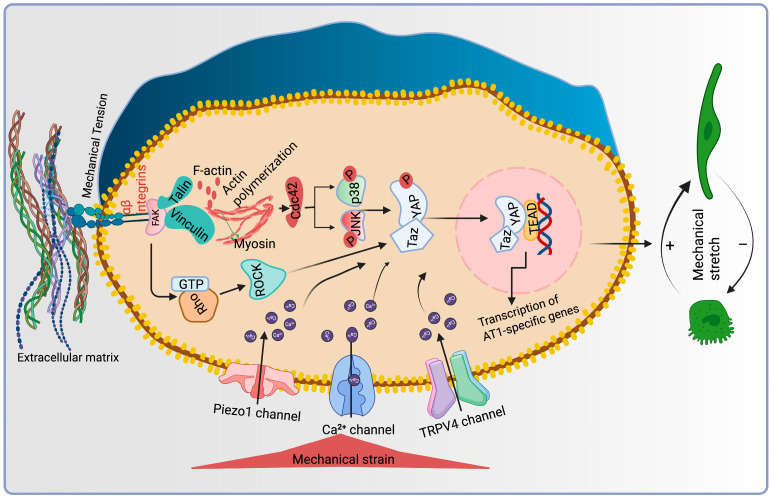
Mechanotransduction-mediated intracellular signaling plays an unwavering mechanistic role in AT2-AT1 differentiation. Mechanical forces generated from fluid pressure and shear stress act on the ECM, which transduces physical force to integrins present on the cell surface. Mechanosensitive focal adhesion proteins allow the integrin clustering, actin polymerization, and cytoskeletal remodeling that transmit forces upon the nuclear envelope, remarkably altering gene transcription. Furthermore, intracellular pathways are activated by the mechanotransduction process. Rho/ROCK and YAP-TAZ are central signaling that activate the transcription of AT1-specific genes. Mechanosensitive channels activate upon stress stimuli, increase the flow of Ca^+^ ions, and simultaneously regulate the YAP/TAZ nuclear shuttling, essential for AT1 cell phenotype.

YAP/TAZ are highly regulated transcriptional co-regulators of the Hippo family that predominantly bind to enhancer sites through DNA-binding proteins named as Tea domain transcription factors (TEAD) [79]. This association was first identified and functionally confirmed in *Drosophila melanogaster* [80]. Numerous cellular inputs are governed by YAP and TAZ, where the phosphorylation of LATS1/2 kinases are triggered by upstream kinases, MST1/2, which phosphorylate and inactivate YAP and TAZ and sustain their cytoplasmic retention and degradation [81]. Nevertheless, a plethora of new research has suggested that cellular mechanotransduction, which predominantly functions in a LATS-independent fashion, is a major factor governing the regulation of YAP/TAZ. Interestingly, YAP/TAZ senses various biomechanical cues and transduces them into biological effects [82], making them universal mechanotransducers and mechanoeffectors. Activated YAP/TAZ accumulates in the nucleus; therefore, the subcellular distribution of these proteins is the fundamental layer of regulation. Cell shape, ECM substrate rigidity, topology, and shear stress are the factors that influence their activation [82,83,84,85]. Activation of YAP/TAZ occurs at elevated mechanosensing levels, e.g., cells are grown on a rigid matrix and are contrary inactivated when cells are rounded and grown on a soft matrix (Matrigel). Similarly, AT2 cells spontaneously differentiated into AT1 cells when grown on a stiff surface and retained AT2 fate when cultured on a soft matrix [86,87]. Recent findings have speculated that YAP/TAZ is necessary for AT2-AT1 differentiation during repair [88]. However, Yap-deficient mice are embryonically lethal, whereas TAZ-KO survives embryogenesis, although it exhibits enlarged alveolar spaces across the lung [89]. During prenatal development, differential pressure in the immature alveoli exhibited relative variations in the AT1/AT2 cells ratio. High amniotic fluid pressure favors AT1 cell differentiation, while low pressure promotes AT2 cell differentiation. Possibly, higher pressure exerted on developing cells promotes YAP nuclear shuttling through elevated cellular stretch, reinforcing AT1 cell development. Moreover, deletion of YAP/TAZ in AT1 cells mediates spontaneous reprogramming into the AT2 cell lineage [90]; on the contrary, the loss of YAP/TAZ displays attenuated AT2 proliferation after bacterial infection in the lungs [91] (Figure 4).

### 11.3. MAPK (ERK1/2, JNK and p38) Signaling

YAP/TAZ is a well-established factor that responds to mechanical signals and reinforces AT1-AT2 trans-differentiation in homeostasis and injury conditions [89,91]. The MAPK pathway has been discovered as a critical component of mechanotransduction. It has been observed that elevated mechanical stress triggers the activation of MAP kinase pathway members, including ERK, p38, and JNK, leading to gene expression and cellular behavior alteration in alveolar cells. These findings underscore the vital participation of the MAPK pathway in translating mechanical signals into biochemical responses within alveolar cells, highlighting its significance in the mechanotransduction process. Growing studies have demonstrated the stemness and self-renewal efficiency of AT2 cells in adult lungs and subsequent differentiation into AT1 cells during injury conditions [92,93,94]. In vivo data show elevated levels of MAPKs under stretch conditions in AT2 cells, followed by activation and nuclear translocation of YAP protein which is essential for AT1 differentiation. Incubation with these MAPK inhibitors suppressed their phosphorylation and subsequently halted the YAP nuclear translocation in stretched AT2 cells, suggesting the suppressed differentiation of AT2 into AT1 cells, ultimately restricting the alveolar repair. These findings conclude the essential role of MAPK activation during the mechanically induced transdifferentiation of AT2-AT1 cells during lung injury [95].

## 12. Nuclear Relocation Acts as a Crucial Regulator for AT1-AT2 Differentiation

The mechanical information triggered through the ECM modifications, perceived by the focal adhesion proteins and transmitted at the cytoskeleton level, affects proteins that are located at the membrane or in the cytoplasm. This information causes proteins to experience structural modifications, which allows them to be shuttled to the nucleus. Several proteins are identified for shuttling across the nuclear membrane in response to mechanical cues. ZO-1 was the first identified protein to shuttle across the nuclear envelope in response to mechanical signals. Nuclear localization of AT1 and AT2-specific proteins and genes defines their cell fate and differentiation during developmental stages, homeostasis, and injury. YAP/TAZ are members of the Hippo signaling. The intracellular and nuclear localization is a key determinant of signal transduction in alveolar cell fate determination. During the developmental phase, mechanical pressure generated from amniotic fluid regulates the actin cytoskeleton in response to cellular stretch and mediates YAP translocation into the nucleus. Subsequently, promotes the increased number of AT1 cells compared to the AT2 cells, as nuclear YAP is the characteristic feature of AT1 cells. Another study reported that mechanical stress in AT2 cells activates YAP and potentiates its nuclear shuttling, determining the AT1 cell fate, promoting alveolar regeneration during injury [95]. Importantly, the loss of YAP/TAZ efficiently drives reprogramming of AT1 into AT2 cells, revealing the significance of nuclear shuttling in sustaining the molecular barrier between the two lineages [90].

## 13. Pathological Mechanical Stress Triggers the Pathological Condition

### 13.1. Ventilator-Induced Lung Injuries

Non-physiological stretch exposure deteriorates the physiological mechanical stress response mechanism and prompts undesired tissue repair, e.g., mechanical ventilation-induced lung injury. AT1 cells perceive mechanotransduction cues via small plasma membrane invagination called caveolae and respond against mechanical stress; however, AT2 cells are devoid of these caveoli [96]. Following mechanical ventilation, the epithelial sodium channel, ENaC, is abnormally activated during the AEC monolayer stretches, which causes a TRPV4-dependent Ca^2+^ wave. The Ca^2+^ wave and other downstream signaling events are likewise blocked by the TRPV4 agonist, GSK1016790A. Moreover, ventilation-induced stretch response triggers Ca^2+^ influx through Piezo1-activated pannexin-1 hemichannels present on caveolae of AT1 cell membrane and stimulates ATP generation. Simultaneously, the release of ATP triggers the secretion of Ca^2+^ from neighboring AT2 cells, creating a hallway for inflammatory mediators [97]. In AT2 cells, mechanical stretch promotes early apoptosis and IL-8 release [98]. Moreover, mechanical stretch increases glucose-regulated protein (GRP78) and activating transcription factor 4/6 (ATF4/6) markers of endoplasmic reticulum (ER) stress, elevating chemokines [99]. Induction of cyclic stretch in AT1 cells promotes ROS production, escalating monolayer permeability through NF-κB and ERK signaling upregulation [100]. Likewise, the AT2 cell monolayer generates ROS such as NO and superoxide under the influence of aberrant mechanical stress [101]. Accumulated ROS mediates FasL/Fas extrinsic death signaling in AT2 cells in newborn rats [102]. Additionally, aberrant mechanical stress enhances the cellular communication network factor 1 (CCN1) in AT1 cells in acute lung injury, impacting tissue responses under non-physiological mechanical stress [103]. Likewise, a recent study indicated that undesired mechanical microenvironments influence AT2-AT1 cell differentiation and fate maintenance, which is an indispensable aspect of lung regeneration [9].

### 13.2. Pulmonary Fibrosis

Pulmonary fibrosis is characterized by excessive fiber deposition in the lung parenchyma due to an aberrant tissue repair process. This process is driven by the alveolar epithelium remodeling, including abnormal ECM deposition. A progressive feed-forward loop is established by compositional and mechanical alterations, wherein escalated matrix deposition supports the differentiation of fibroblasts, which sustains matrix synthesis and stiffening. Mechanotransduction pathways sense and respond to the biomechanical features of tissues, enabling cells to detect changes in the mechanical cues and translate mechanical information into biochemical signals downstream [104]. AT2 cells exhibit stem cell activity due to their ability to differentiate into AT1 cells during lung injury and mediate the regeneration and repair process. Thus, the impaired renewal and differentiation ability of AT2 governs fibrogenesis and secretion of pro-fibrotic factors [105]. TGF-β1 signaling is activated by mechanical stress in the AT2 cells, which changes the optimal microenvironment in the lung, stimulating the abnormal repair [106]. Tissue mechanical stress-mediated TGF-1 activation is regulated by Rho/ROCK signaling cascade via interaction with surface integrins and FAK proteins [107]. These interactions dysregulate the functions of AT2 cells and subsequently retard the self-renewal and differentiation ability into AT1 cells essential for adequate tissue repair and regeneration leading to fibrosis progression [108]. TGF-β1 signaling in AT2 cells is stimulated by non-physiological mechanical stress, resulting in progressive lung fibrosis that extends from the periphery to the center. A recent study indicated that AT2 could be the source of TGF-β, which activates lung fibroblasts [106] in mechanical stress. Additionally, Tgfb1 shRNA intervention substantially decreases collagen expression levels in stromal cells and Tgfb1 expression in AT2 cells. Loss of CDC42 in AT2 cells is essential to maintain proliferative capacity. Via activation of the CDC42/F-actin/MAPK/YAP signaling, YAP promotes regeneration and fibrotic lesion regression while suppressing inflammation [95]. Since abnormal tissue repair is the primary cause of fibrosis, damaged AECs’ integrity catalyzes the fibrotic response [109]. Mechanical stress triggers oxidative damage, ER stress, and the release of injury molecules, such as DAMPs, which signal the commencement of a fibrotic response and the repair of tissues [110]. In conclusion, under non-physiological mechanical stress, impaired AT2 cells exhibit impaired functional activity manifested by mitigated AT1 differentiation and release of vital pro-fibrotic factor, which promotes fibrotic responses.

## 14. Conclusive Remark and Future Direction

Mechanotransduction is fundamental to alveolar cell development and differentiation, acting as a critical interface between the physical environment and cellular signaling. During fetal development, mechanical forces such as cyclic stretch and fluid sheer stress from fetal breathing movements and amniotic fluid pressure activate various mechanosensitive pathways (FGF, YAP/TAZ, RhoA/ROCK, and FAK) and ion channels (TRP and Piezo), promoting alveolar cell proliferation and differentiation (AT2 to AT1 transition), and supporting lung architecture and function. These mechanisms are integrated with biochemical cues (FGF signaling) to ensure the robust and proper alveolation.

In pathological conditions such as mechanical ventilation and fibrosis-induced matrix stiffness deregulated the mechanical forces (excessive or aberrant). This leads to maladaptation in cellular responses, such as impaired alveolar differentiation and persistent intermediate cell state. The dysregulated condition underlies various chronic and acute lung pathologies, highlighting the dual nature of mechanotransduction as both a driver of resilience and vulnerability of the lung.

In the future, immense amounts of research are needed to investigate the adverse consequences caused by non-physiological mechanical stimuli on alveolar cells and further elucidate the regulatory mechanisms of these signaling communications. While in vivo studies are essential, sophisticated in vitro models that better recapitulate the complex mechanical environment of the lung are needed. The use of 3D organoid models, microfluidic “lung-on-a-chip” systems, and engineered matrices with tunable stiffness and dynamic stretch capabilities will allow for more precise control and manipulation of mechanical cues. Additionally, integrating single-cell transcriptomics and proteomics with biophysical measurements will provide an unprecedented resolution to understand cell-to-cell heterogeneity in mechanosensing and its impact on cell fate.

A deeper understanding of mechanotransduction could lead to novel therapeutic strategies for lung diseases. By exploring the intensity of aberrant mechanotransduction, which contributes to fibrosis, researchers can delve into novel interventions that robustly prevent excessive ECM deposition and promote healthy tissue repair. This process might involve developing drugs that modulate specific mechanosensing proteins or signaling pathways or designing biomaterials with tailored mechanical properties to guide the alveolar cell fate for regenerative medicine applications.

## Abbreviations

**AT1**	Alveolar type I
**AT2**	Alveolar type II
**CAR4**	Carbonic anhydrase 4
**CCN1**	Cellular communication network factor 1
**ECM**	Extracellular matrix
**FGF10/12**	Fibroblast growth factor 10/12
**FAK**	Focal adhesion protein
**Id2**	Inhibitor of DNA binding 2
**PDGFRB**	Platelet-derived growth factor B
**PTK2**	Protein Tyrosine Kinase 2
**TMEM63A**	Transmembrane protein 63A
**ROCK**	Rho-associated coiled-coil-containing kinases
**YAP/TAZ**	Yes-associated protein (1) and TAZ (transcriptional coactivator with PDZ motif)
**MAPK**	Mitogen-activated protein kinase
**ERK**	Extracellular signal-related kinase
**JNK**	c-Jun N-terminal kinases
**LADs**	Lamina-associated domains
**LINC**	Linker of the nucleoskeleton and cytoskeleton
